# Observations from the first 100 cases of intraoperative MRI – experiences, trends and short-term outcomes

**DOI:** 10.1186/s12893-024-02569-y

**Published:** 2024-09-19

**Authors:** Hanna Barchéus, Christoffer Peischl, Isabella M. Björkman-Burtscher, Christina Pettersson, Anja Smits, Daniel Nilsson, Dan Farahmand, Johanna Eriksson, Thomas Skoglund, Alba Corell

**Affiliations:** 1 Department of Clinical Neuroscience, Institute of Neuroscience and Physiology, Sahlgrenska Academy, University of Gothenburg, Blå stråket 7, Gothenburg, 41346 Sweden; 2 Department of Neurosurgery, Sahlgrenska University Hospital, Region Västra Götaland, Gothenburg, Sweden; 3 Department of Radiology, Sahlgrenska University Hospital, Region Västra Götaland, Gothenburg, Sweden; 4 Department Hybrid and Intervention Operation 5, Sahlgrenska University Hospital, Region Västra Götaland, Gothenburg, Sweden; 5 Department of Radiology, Institute of Clinical Sciences, Sahlgrenska Academy, University of Gothenburg, Gothenburg, Sweden

**Keywords:** Neurosurgery, MRI scan, Brain tumors, Deep brain stimulation

## Abstract

**Background:**

We sought to analyze, in well-defined clinical setting, the first 100 patients treated at the intraoperative MRI (iMRI) hybrid surgical theatre at our facility in a population-based setting to evaluate which pathologies are best approached with iMRI assisted surgeries, as this is not yet clearly defined.

**Methods:**

Patients undergoing surgery in the 3T iMRI hybrid surgical theatre at our neurosurgical department between December 2017 to May 2021 were included after informed consent. Demographic, clinical, surgical, histological, radiological and outcome parameters, as well as variables related to iMRI, were retrospectively collected and analyzed. Patients were subdivided into adult and pediatric cohorts.

**Results:**

Various neurosurgical procedures were performed; resection of tumors and epileptic foci, endoscopic skull base procedures including pituitary lesions, deep brain stimulation (DBS) and laser interstitial thermal therapy (LITT). In total, 41 patients were pediatric. An iMRI scan was carried out in 96% of cases and led to continuation of surgery in 50% of cases, mainly due to visualized remaining pathological tissue (95.2%). Median time to iMRI from intubation was 280 min and median total duration of surgery was 445 min. The majority of patients experienced no postoperative complications (70%), 13 patients suffered permanent postoperative deficits, predominantly visual.

**Conclusion:**

Herein, we demonstrate the first 100 patients undergoing neurosurgery aided by iMRI at our facility since introduction. Indications for surgery differed between pediatric and adult patients. The iMRI was utilized for tumor surgeries, particularly adult low-grade gliomas and pediatric tumors, as well as for epilepsy surgery and DBS. In this heterogenous population, iMRI led to continuation of surgery in 50%. To establish the benefit in maximizing the extent of resection in these brain pathologies future studies are recommended.

**Clinical trial number:**

Not applicable.

## Introduction

The innovation and development of imaging modalities over the last decades have been an essential component in the neurosurgical arsenal, facilitating both diagnostics and treatment of lesions within the intracranial vault [1–6]. Since the introduction of magnetic resonance imaging (MRI) [7], technical advancement regarding configuration, imaging quality and magnetic field strength have enabled subsequent evolvement of the intraoperative MRI (iMRI) [5, 8, 9].

At present, applications for iMRI are numerous [10]. Utilization is notably advantageous for neurosurgical oncology and tumor resection [5, 6, 11], where maximum extent of resection (EOR) is an established primary endpoint [12–20]. When detecting residual tumor on intraoperative images, iMRI facilitates immediate continuation of surgery to maximize EOR [11, 21], and a possibility to adjust for navigation inaccuracies rendered intraoperatively by brain shift [5, 10, 11, 22, 23]. In addition to treatment of primary intra-axial tumors, recurrences, and metastases, iMRI is utilized in treatments of epileptic foci, stereotactic procedures, and neurosurgical conditions within the pediatric population [3, 4, 24]. The latter is additionally favored by iMRI as preceding anesthetics in connection with postoperative MRI is often required in this patient group. Recent evidence suggests that iMRI may indeed mitigate this need [25–27]. Moreover, iMRI permits interventional treatments such as laser interstitial thermal therapy (LITT) [28] and monitoring during perioperative motor mapping or awake mapping of complex cognitive functions [29].

iMRI is sought to reduce inefficiency, provide greater accuracy and maximize resection while minimizing morbidity [7, 11, 21, 25]. An iMRI-customized operating theatre, however, presents with many challenges as three significant forces are present when employing an iMRI; the static magnetic field, the time-varying gradient magnetic field, and the radio-frequency fields [30]. Thus, screening for contraindications and MR safety related patient risks are essential prior to iMRI aided interventions. Further, an iMRI operating theatre requires MRI safe or conditional equipment, sufficient space for surgical and anesthesiologic equipment correctly distanced from the scanner and its stray field, highly specialized visual aid and patient monitoring systems and a thorough safety checklist and regular training of personnel [8, 31]. Additionally, the iMRI procedure itself prolongs operative time, affecting the quantity of surgeries performed in the operating theatre [5, 32, 33]. All aforementioned aspects are costly and high-maintenance features to be considered.

Utilization of iMRI has previously been established in selected patient cohorts, such as gliomas and pituitary tumors [34, 35]. However, few studies have displayed the wide range and versatility of iMRI. Previous investigations emphasize the need for additional studies in well-defined clinical settings to elucidate which pathologies are best approached with iMRI assisted surgeries, as this is not yet clearly defined [5, 36]. In this study, we therefore sought to analyze the first 100 patients treated at the iMRI hybrid surgical theatre at our facility in a population-based setting, as a basis for defining future studies. Associated clinical variables related to the procedures and outcomes were studied, as well as the clinical indications set by the neurosurgeons to utilize iMRI, aiming to identify which conditions are best favored by this state-of-the-art technology.

## Method

### Study population

The iMRI surgical theatre at Region Västra Götaland, Sahlgrenska University Hospital, Gothenburg, Sweden, opened for surgery December 4th in 2017. The neurosurgical department at Sahlgrenska University Hospital favors a catchment area of 1.8 million inhabitants. The first 100 patients who underwent neurosurgery at the iMRI hybrid facility and consented to participation in the study were included. In total, 16 patients refrained from inclusion due to declined participation or lack of consent owing to other circumstances. The inclusion period stretched between December 2017 and May 2021.

### Clinical variables

Clinical patient data and demographics were obtained through retrospective analysis of electronic health records (EHR). Variables studied included patient outcome, peri- and postoperative complications, as well as neurological performance in the follow-up period of a minimum of 3 months, to assess deficits to be transient or severe. Postoperative outcome was registered within 30 days according to the Landriel-Ibañez classification system, ranging from grade 1 to 4 with a subdivision of surgical or medical complications [37]. Preoperative neurological deficits were acquired from EHR. Neurological deficits after surgery were divided into new or worsened deficits, subdivided into minor or severe. Severe neurological deficits were classified as those severely impacting life (aphasia/severe aphasia, motor function grade < 4 on Medical Research Council (MRC)) based on EHR or cognitive deficit affecting daily life [38, 39]. Severe cognitive deficits affected daily life and ability to work. Neurological deficits persisting more than 3 months were considered permanent.

### Clinical decision making to utilize iMRI

The clinical indications by the neurosurgeons for choosing iMRI were defined as:

1. Rationalizing pre-operative MRI work up (including anesthesia) in the pediatric, respective adult, population.
2. Obtaining peri-operative stereotactic coordinates for DBS patients without peri-operative transportation of patient under anesthesia.
3. Safely maximizing EOR in pediatric and adult populations.
4. Obtaining postoperative images to assess end result without additional anesthesia for the pediatric population.

### Intraoperative MRI

**Fig. 1:**
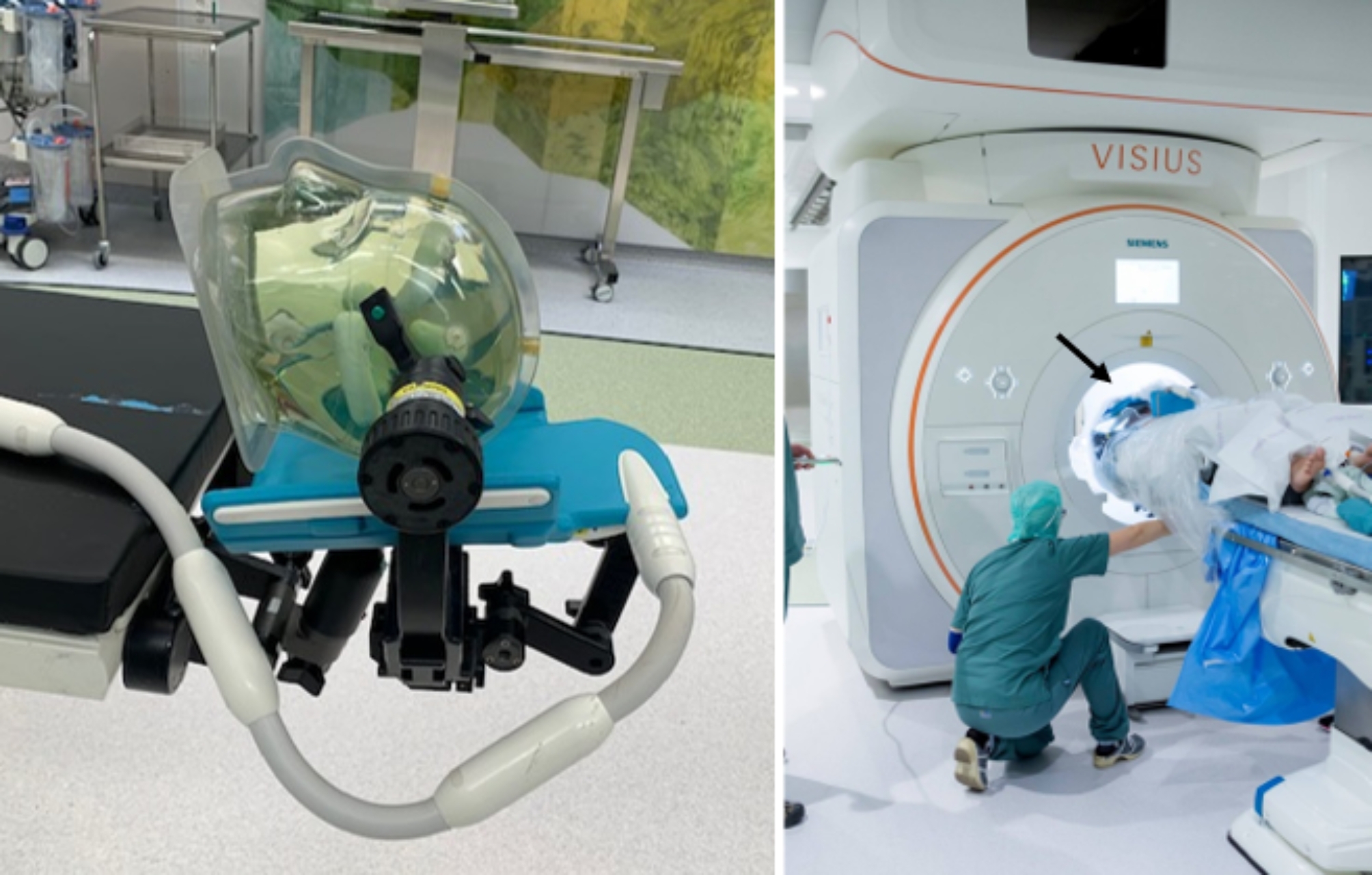
iMRI suite set up. **Left**: Patient model with affixed Mayfield charcoal skull clamp and one 4-channel receiver flex coil (blue) below the head. **Right**: Intraoperative positioning of sterile dressed patient on the fixed operating table with the second receiver flex coil in blue (black arrow) placed over the patients’ head after temporary closure of surgical wound. The patient head is positioned in the center of the bore during iMRI scanning

The iMR suite (IMRIS Visius Surgical Theatre, IMRIS Inc., Winnipeg, Canada) at our hybrid surgical facility employs a modified ceiling-mounted 3T movable MR scanner (MAGNETOM Skyra; Siemens Healthineers, Erlangen, Germany; software release VE11C). When not utilized in surgery, the scanner is positioned in an adjoining garage, separated from the surgical theater by a radiofrequency-shielded and semi sound shielded sliding door. This allows operation of the MR scanner for diagnostic and research purposes while surgery is proceeding in the surgical theatre. The 0.5 mT line of the stray field reaches 2 m into the surgical theatre. Due to the size of the surgical theater, the 0.5 mT line does not affect the area where the patient is positioned during surgeries. However, safety routines for the least active device needs to be rigorously followed at all times and by any person entering the surgical theatre, even when the IMRIS scanner is parked in the garage with doors closed. See Fig. 1 for set-up of the iMRI suite.

Imaging was performed using the transmit body coil and two 4-channel receiver flex coils (HC300, IMRIS, Deerfield Imaging Inc), placed under and above the head. Prior to surgery, patients were in the surgery theater position on a dedicated operation table with the head (cervical spine in one case) resting on the lower flex coil. Adequate patient positioning considering the coil, the Mayfield charcoal fiber scull clamp (HFD100, IMRIS, Deerfield Imaging Inc), the neuronavigational arm, and the surgical area was established by threading a circular model of the scanner bore opening (VISIUSeye, IMRIS) over the patients’ head. For children under one year, a gel or vacuum cushion was utilized instead of the Mayfield skull clamp. The sterile dressing was then placed and surgery initiated. Intraoperative imaging required temporary closure of the wound and enclosing the head with a sterile plastic cocoon. The upper flex coil was placed over the patients head outside of the cocoon. Before the MR scanner was brought into the operating theatre, it was made sure that all devices were according to their MR labeling (MR safe, conditional or unsafe) positioned in marked zones within the theater. The procedure was in accordance with the safety protocol and finalized with a hard stop declared by the MR safety nurse.

Pre-, peri- and post-operative MR protocols for brain imaging included, depending on lesion type to be visualized, the following imaging sequences: T1-weighted (T1w) 3D MPRAGE (Magnetization Prepared Rapid Gradient Echo) acquired with and/or without gadolinium (Gd) contrast medium (Clariscan^®^ 0.2 ml/kg body weight); T2w 3D FLAIR (fluid attenuation inversion recovery); T2w 2D SE (spin echo) in axial and/or coronal plane which was rarely substituted by a T2w 3D SE sequence; DWI (diffusion weighted imaging). Direct assessment of residual pathological tissue was conducted by a consulting neuroradiologists in cooperation with neurosurgeons in the operating theatre, imperative to minimize excess time in the OR as well as time of anesthesia. When the scanner was repositioned in the garage, surgery was continued if deemed necessary.

### Radiological variables

Radiological variables were retrieved from EHR and comprised location of lesion, size, and intraoperative findings. Intraoperative residuals of lesions were evaluated primarily on FLAIR or T2w images for non-enhancing tumors, contrast enhanced T1w images for enhancing tumors, and the sequence with best lesion visualization in epilepsy surgery.

### Surgical variables

Information about surgical procedure, tissue sampling, histopathological diagnosis and surgical outcome were obtained through EHR. Planned surgical aim, that is, EOR (gross total resection [GTR] or subtotal resection [STR]) was extracted from the surgeon’s assessment of individual patients EHR. GTR was defined as no visible tumor left on the iMRI sequence on which the lesion was best identified preoperatively. Surgical adjuncts such as navigation, microscope, ultrasonic aspirator and ultrasound were logged. Time of surgical procedure was measured from start of anesthesia to extubation. The surgical rationale for utilization of iMRI aided surgeries were retrieved through interviews with neurosurgeons. Surgical data are demonstrated in Table 1.

**Table 1:** Surgical characteristics for the complete cohort (*n* = 100) and age-dependent subgroups (pediatrics, *n* = 41; adults *n* = 59)

Variables	Patients, *n* = 100	Pediatrics, *n* = 41	Adults, *n* = 59	*P*-value
**Surgical aim, ** ***n *** **patients (%)**				
Gross total resection	68 (68.0)	32 (68.0)	36 (61.0)	0.07
Subtotal resection	15 (15.0)	5 (12.2)	10 (16.9)	0.51
**Surgical procedures**,** n patients (%)**				
Intracranial tumor craniotomy	60 (60.0)	31 (75.6)	29 (49.2)	0.08
Endoscopic skull base including sella	18 (18.0)	1 (2.4)	17 (28.8)	**< 0.01**
Deep brain stimulation	9 (9.0)	0 (0)	9 (15.3)	**0.009**
Resection epileptic focus	7 (7.0)	6 (14.6)	1 (1.7)	**0.013**
Laser ablation	3 (3.0)	0 (0)	3 (5.1)	0.14
Biopsy	2 (2.0)	2 (4.9)	0 (0)	0.09
Extirpation tumor in spinal canal	1 (1.0)	1 (2.4)	0 (0)	0.23
**Surgical duration**,** (minutes) median (SD)**				
Time to MRI	280 (130)	275 (105)	295 (147)	0.69
Total time of surgery	445 (150)	420 (127)	471 (163)	0.29

### Statistical analysis

IBM SPSS Statistics software program version 24.0 was used to perform statistical analyses. Data were presented as count values, proportions in percentage, means, and medians. Spread was presented with SD, apart from age which was presented as range, with a significance level of *p* ≤ 0.05. Normal distribution was investigated with histogram and Kolmogorov-Smirnov test. For comparison of postoperative deficits in subgroups, a chi-square test was utilized. Comparison of continuous variables were conducted using independent sample t-test for normally distributed data, and Mann-Whitney U-test for skewed data.

## Results

### Cohort characteristics

We enrolled 100 patients where 42 (42%) were female. The mean age was 32 years (range 0–81 years). Patients were divided into two cohorts depending on age; adults (*n* = 59) and pediatric patients (*n* = 41), where mean age was 48 years (range 18–81 years) and 9 years (range 0–17 years) respectively. Lesions were predominantly located in the frontal lobe (24.5%) in adult patients and in the skull base, including sella and sinonasal (30.5%) in pediatric patients. They were right sided in 33% and located in the midline in 29% of patients. Mean size of lesion was 31.0 mm (SD 16.3) in all patients. For more details on clinical characteristics, see Table 2.

**Table 2:** Clinical characteristics for the complete cohort (*n* = 100) and age-dependent subgroups (pediatrics, *n* = 41; adults *n* = 59)

Demographics	Patients (*n* = 100)	Pediatrics (*n* = 41)	Adults (*n* = 59)
Age at surgery (years), mean (SD)*	32 (23.3)	9 (5.2)	48 (16.3)
Female, n (%)	42 (42.0)	19 (46.3)	23 (39.0)
**Main lesion location**,** n patients (%)**			
Frontal	21 (21.0)	10 (24.5)	11 (18.6)
Temporal	15 (15.0)	3 (7.3)	12 (20.3)
Parietal	5 (5.0)	2 (4.9)	3 (5.1)
Occipital	4 (4.0)	3 (7.3)	1 (1.7)
Insular	2 (2.0)	1 (2.4)	1 (1.7)
Cerebellar	7 (7.0)	6 (14.6)	1 (1.7)
Skull base, sellar and sinonasal	20 (20.0)	2 (4.9)	18 (30.5)
Deep brain structures	6 (6.0)	4 (9.8)	2 (3.4)
Ventricular	6 (6.0)	6 (14.6)	0 (0.0)
Other**	5 (5.0)	4 (9.8)	1 (1.7)
Not applicable, DBS electrode placement	9 (9.0)	0 (0.0)	9 (15.3)
**Size**			
Largest diameter (mm), mean (SD)	31.0 (16.3)	25.6 (13.6)	35.4 (17.1)

*Rounded down or up to full year. **Including opticus (*n* = 2), cervical spinal medulla (*n* = 1), chiasma (*n* = 1) and brainstem (*n* = 1)

### Surgical strategy

The most prevalent surgical aim was GTR (68%) in both adult and pediatric patients. The majority of patients underwent surgery in the supine position (91%), the remainder underwent surgery in prone positioning (9%). Intracranial tumors constituted indications for surgery in 75.6% of children and 49.2% of adults (*p* = 0.08). In the adult cohort, 17 (28.8%) patients underwent transsphenoidal skull base surgery compared to 1 (2.4%) in the pediatric cohort (*p* < 0.01). Significantly more patients in the pediatric cohort underwent resection of an epileptic focus (14.6%) compared to adults (1.7%) (*p* = 0.013). DBS was exclusively performed in adult patients (9%). Median time to iMRI from start of anesthesia and median time of surgery for the whole cohort was 280 min (SD 130) and 445 min (SD 150) respectively. For details, see Table 1.

### Intraoperative variables

An iMRI scan was performed in 96% of cases. IMRI was initially planned, but refrained from, in 4 out of 20 patients with skull base pathologies during the first time period after implementation. Continuation of surgery after iMRI was performed in 54 patients (56.3%). However, continuation of surgery after iMRI was not always part of the expected procedure, as DBS and LITT constitute special surgical circumstances. In the 9 patients undergoing DBS (9%), an iMRI scan was exclusively utilized for surgical planning, as additional intraoperative scanning after placement of electrodes was unfeasible due to, at the time, unapproved systems for the 3T MRI scanner. Three patients underwent treatment with LITT (3%), where the near real time surveillance of the iMRI is an essential part of intervention, rather than a postoperative evaluation of surgical results. For details regarding surgical procedures and duration of surgery, see Table 1.

**Fig. 2:**
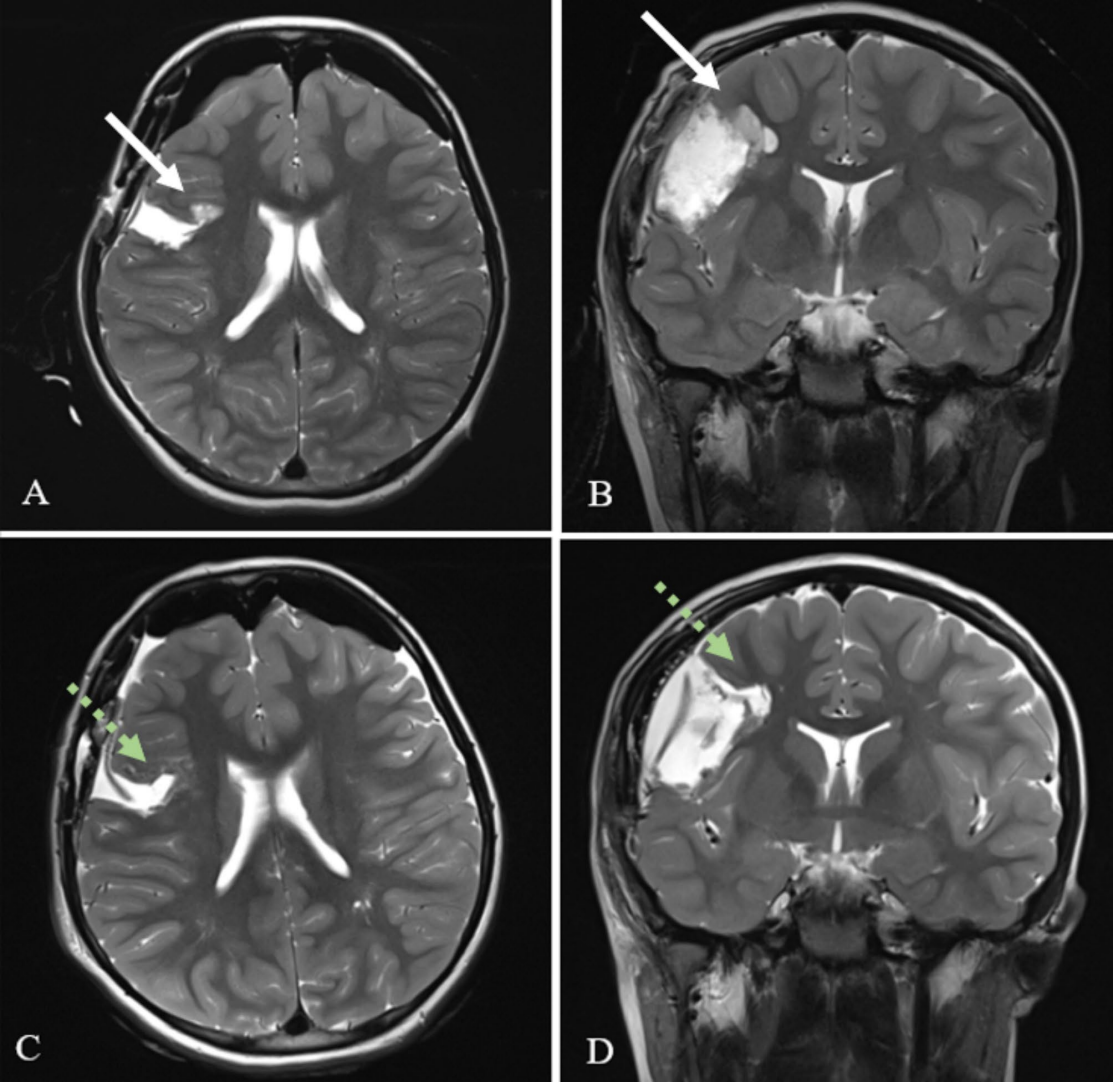
Intraoperative T2-weighted MRI images of a dysembryoplastic neuroepithelial tumor (DNET) in the right frontal lobe before (**2 A-B)** and after (**2 C-D)** continued resection following iMRI. Remaining tumorous tissue detected on iMRI before extended resection is demonstrated with white arrows (**2 A-B).** After resection, achieved GTR is demonstrated with green arrows (**2 C-D)**

**Fig. 3:**
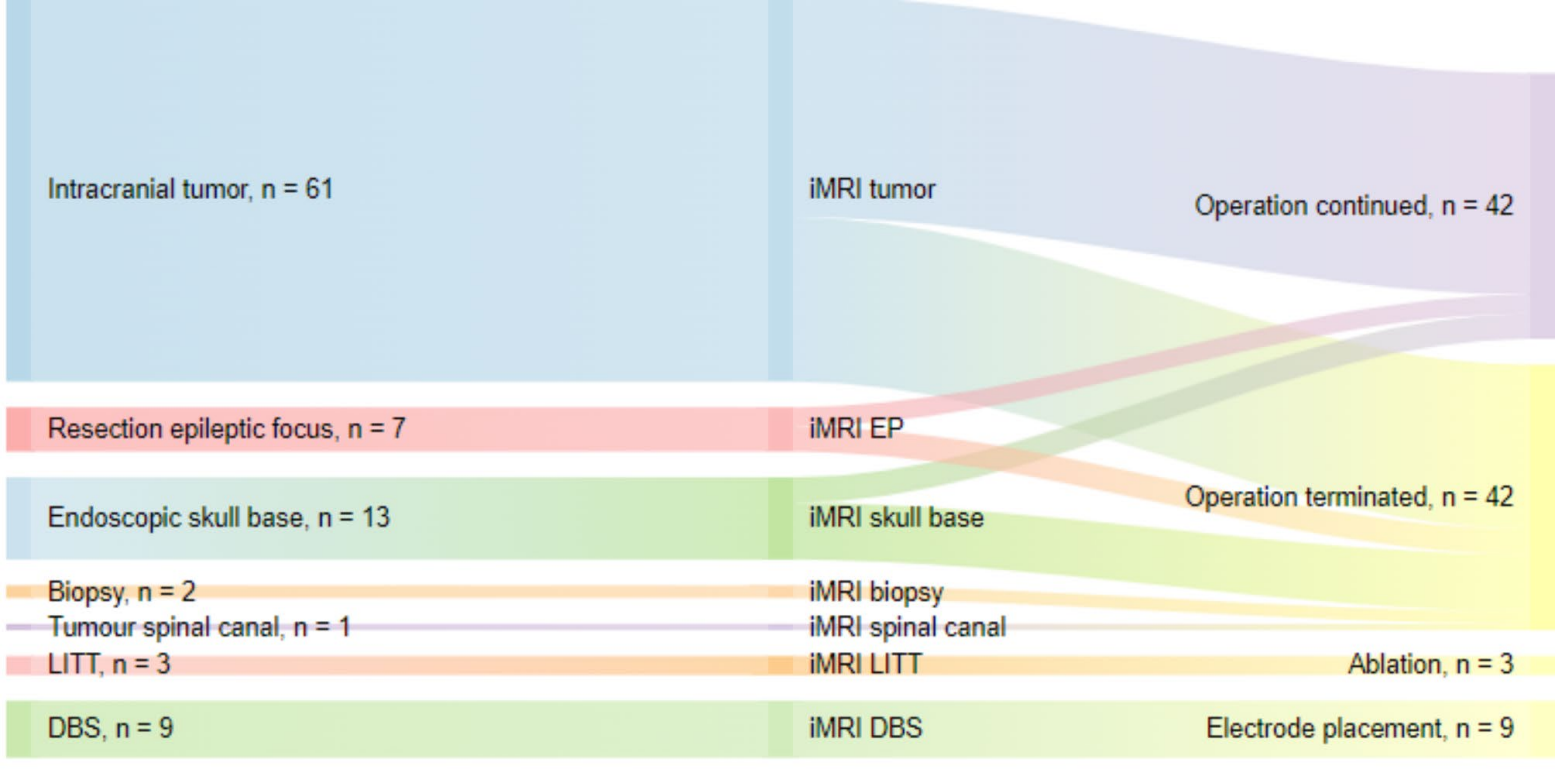
Sankey plot over performed surgeries and whether surgery was continued or not (*n* = 96). Patients undergoing endoscopic skull base surgeries with pituitary/skull base lesions who did not perform an intraoperative MRI (4) are excluded in Fig. 3

Out of the remaining 84 patients, 42 surgeries (50%) recommenced surgery after the iMRI, predominantly due to visualized remaining pathological tissue (95.2%), where the vast majority consisted of remaining neoplastic tissue (90.5%). See Fig. 2 for visualization of intraoperative imaging before and after continued resection. Two surgeries were resumed due to remaining epileptogenic tissue (4.6%) and one surgery was continued for inspection. One surgery was resumed to manage an acute hematoma discovered on intraoperative images, in addition to which there were two other intraoperative events recorded; one intraoperative seizure during awake surgery, and one surgery was paused temporarily due to an increase of lactate in blood. Out of the 42 patients where surgery was terminated after the iMRI, 38 cases (45%) were discontinued as surgical aim was already achieved (GTR, STR or biopsy). In four cases (9.5%), surgery was discontinued despite GTR not being reached. For depiction of course of actions, see Fig. 3 below.

Surgical adjuncts such as ultrasound were applied in 28.8% of adults and 12.2% of pediatric patients. Neuronavigation was utilized in 76.3% of adults and 70.7% of pediatric patients. Motor mapping was exercised in 13.6% of adults and 9.8% of pediatric patients. Awake mapping and 5-aminolevulinic acid (5-ALA) were utilized in only 1 (1.7%) adult respectively. Tissue samples were obtained in 91% of patients (all but 9 patients whom received DBS). A variety of histopathological diagnoses were identified, see Table 3 below.

**Table 3:** Histopathological diagnosis of total patient cohort (*n* = 90) * and age-dependent subgroups (pediatrics, *n* = 41; adults *n* = 49)

Histopathological diagnosis, *n* patients (%)	Patients, *n* = 90	Pediatrics, *n* = 41	Adults, *n* = 49
Astrocytoma grade 1–4	32 (35.6)	15 (36.6)	17 (34.7)
Pituitary adenoma	11 (12.2)	1 (2.4)	10 (20.4)
Oligodendroglioma	6 (6.7)	0 (0.0)	6 (12.2)
Focal cortical dysplasia	5 (5.6)	4 (9.8)	1 (2.0)
DNET**	4 (4.4)	3 (7.3)	1 (2.0)
Ependymoma	4 (4.4)	4 (9.8)	0 (0.0)
Chordoma	3 (3.3)	0 (0.0)	3 (6.1)
Craniopharyngioma	3 (3.3)	2 (4.9)	1 (2.0)
Gliosis	3 (3.3)	3 (7.3)	0 (0.0)
Ganglioglioma	2 (2.2)	0 (0.0)	2 (4.1)
Medulloblastoma	2 (2.2)	2 (4.9)	0 (0.0)
Papilloma	2 (2.2)	2 (4.9)	0 (0.0)
Meningioma	2 (2.2)	1 (2.4)	1 (2.0)
Other***	11 (12.1)	4 (9.8)	7 (14.3)

*In one adult, lack of representative material led to inconclusive tissue diagnosis. **Dysembryoplastic neuroepithelial tumor. ***One case of each of the following were detected in pediatric patients (2.4%): Tuberous sclerosis, teratoma, cavernoma and subependymal giant cell astrocytoma (SEGA). In adults, one undifferentiated sinonasal lesion, limbic encephalitis, Langerhans cell histiocytosis, fibrous dysplasia, pineal parenchymal tumor of intermediate differentiation (PPTID), neuroblastoma and hamartoma was detected (2.0%)

### Postoperative outcome

Postoperative complications were recorded within 30 days after surgery and classified according to the Landriel-Ibañez classification [37] (Table 4). The majority of patients suffered no postoperative complications (70%) and there were no fatalities within 30 days. Postoperative motor deficits were observed in 11%, visual impairment in 10%, language deficit in 8%, cognitive deficit in 5% and cranial nerve palsy in 4% of all patients. In adults, all motor and language deficits were transient, whereas cranial nerve deficits were permanent in all adults and in 50% of pediatric patients. In total, 14 postoperative deficits were permanent in 13 different patients. There was no significant difference in frequencies of post-operative deficits between cases, irrespective if surgery was continued after iMRI or not (*p* = 0.67). Visual deficits were permanent in 83.3% of adults and in 50% of pediatric patients with postoperative visual deficits. Children did not experience any cognitive deficits. For details regarding severity and if permanent or transient, see Table 4.

**Table 4:** Postoperative variables including complications according to Ibanez classification and postoperative deficits (*n* = 100)

	Patients	Pediatrics	Adults
**Complications according to Landriel-Ibañez,** ***n*** **patients (%)**			
	***n*** ** = 100**	***n*** ** = 41**	***n*** ** = 59**
No complication	70 (70.0)	27 (65.8)	43 (72.9)
Ia (no drugs)	9 (9.0)	5 (12.2)	4 (6.8)
Ib (drugs)	13 (13.0)	6 (14.6)	7 (11.9)
IIa (intervention without general anesthesia)	2 (2.0)	0 (0.0)	2 (3.4)
IIb (intervention with general anesthesia)	3 (3.0)	2 (4.9)	1 (1.7)
IIIa (single organ failure requiring ICU)	3 (3.0)	1 (2.4)	2 (3.4)
IIIb (multiorgan failure requiring ICU)	0 (0.0)	0 (0.0)	0 (0.0)
IV (death)	0 (0.0)	0 (0.0)	0 (0.0)
**Type of complication**,** n patients (%)**			
	*n* = 30	*n* = 14	*n* = 16
Medical	19 (63.3)	8 (57.1)	11 (68.8)
Surgical	11 (36.7)	6 (42.9)	5 (31.3)
Postoperative deficits*,** n patients (%)**			
	*n* = 29	*n* = 11	*n* = 18
Motor	11	5	6
• Severe	2	1	1
• Permanent	1	1	0
Language	8	2	6
• Severe	1	0	1
• Permanent	0	0	0
Cognitive	5	0	5
• Severe	2	0	2
• Permanent	3	0	3
Visual	10	4	6
• Severe	3	1	2
• Permanent	7	2	5
Cranial nerves	4	2	2
• Severe	2	1	1
• Permanent	3	1	2

*More than one deficit could occur per patient. IIa complications included leakage of cerebrospinal fluid (*n* = 2), IIb included surgery for infected bone flap (*n* = 1), cerebrospinal fluid fistula (*n* = 1) and subgaleal accumulation (*n* = 1), all of which assed as surgical complications. IIIa complications included syndrome of inappropriate antidiuretic hormone (SIADH) and cortisol deficiency (*n* = 1, medical), hygroma (*n* = 1, surgical) and focal and tonic-clonic generalized seizures (*n* = 1, surgical)

## Discussion

Herein, we present our experiences and findings from the first 100 patients undergoing neurosurgical procedures aided by the iMRI at our facility. The aim of our study was to characterize the variables of this patient population, as a basis for determine the role of iMRI in specific brain pathologies in future studies. In all patients, intracranial tumors constituted main reason for surgery, indicating a beneficial role of this state-of-the art treatment in cases of tumor surgery. In our experience, this is especially true for suspected low-grade tumors, an experience supported by Levy et al. for the pediatric population [40]. Furthermore, utilization of iMRI appears to be beneficial in cases of epilepsy surgery and DBS.

As previously stated, gliomas constituted main indication for surgery in the iMRI theatre at our facility, for which the surgical endpoint is to maximize EOR without inflicting permanent neurological deficits, in accordance with the clinical rationale for choosing iMRI-aided surgeries [5, 12–20, 41, 42]. The clinical implication to maximize EOR is not limited to the diminished need of a postoperative MRI, or reoperation due to remaining tumor or epileptic foci, but further extends to the gain for the individual patient [43]. However, due to the limited accessibility of the hybrid operating theatre, our neurosurgeons found it to be most valuable in resection and extirpation of suspected lower-grade tumors, as these neoplasms exhibits a slower growth rate and hence surgeries planned far in advance are feasible. Although we cannot draw any conclusions from these data, a subanalysis of nine patients included in the cohort with glioma WHO grade 2 demonstrated that a median of 97,55% extent of resection (EOR) was reached (data not shown). For GBM and HGG patients, previous studies demonstrate that usage of iMRI resulted in longer progression free survival (PFS), a valuable time for patients with this devastating diagnosis [42, 44]. Due to the rapid growth rate demonstrated by GBMs, patients with suspected GBM shall be operated within two weeks of radiological diagnosis, in accordance with the Swedish standardized care pathways; A time frame that is not logistically possible to adhere to within the iMRI hybrid theatre at out clinic. Unfortunately, this results in inequalities in the patient care between different patient groups.

From a resource- and patient perspective, utilization of iMRI seems to be further beneficial in pediatric patients. One major advantage of iMRI is that it minimizes the need for an additional postoperative iMRI, which in most pediatric patients requires general anesthesia or sedation. A meta-analysis performed by Wach et al. regarding the impact of iMRI in pediatric brain tumor surgery concludes that iMRI-guided surgery appears to improve EOR in pediatric glioma surgery, while maintaining similar frequencies of new neurological deficits comparable to other procedures [45]. These observations are further supported by Wu et al. who, somewhat more tentatively, states that iMRI potentially may aid surgeons in achieving maximum EOR in pediatric brain tumor patients [46]. A recent study by Avula et al. demonstrates that with the improved image quality of the 3T-scanner, there is no difference between iMRI and early- postoperative images (24–72 h) regarding residual tumor margin amogst the pediatric population [27], a finding which potentially negates the need for an additional postoperative scan. Furthermore, in our experience, the neurosurgical threshold for continuing the resection is lower when the patient is still in the operating theatre compared to when patients have awoken from anesthesia in the department. Consequently, patients would to a lesser extent hence have to undergo reoperations owing to remaining pathological tissue. This theoretical advantage is supported by Choudhri et al. who demonstrated a significant decrease in early reoperation rates after the introduction of iMRI in their clinic, reducing reoperations from 8 to 1% amongst their patients [47].

However, other indications beyond tumor surgery, such as DBS and LITT, should not be overlooked. An iMRI with ceiling mounted design ensures higher security for the surgical procedures as it allows the patient to remain in the operative position throughout the surgery, including when the iMRI is performed, thus minimizing the risk of complications associated with perioperative transportation. This is particularly beneficial for patients undergoing surgery for DBS electrode placement, as patients otherwise are put under anesthesia before the stereotactic frame is fixed onto the patients’ head. Patients are then transported to the MRI scan where images are obtained, before being transported back to the operating suite for commencement of surgery. For DBS-patients, employment of the iMRI reduces the risk of complications associated transportation under anesthesia, as well as reduces time of surgery. Regarding LITT-treatment, the near real-time surveillance of the iMRI is a fundamental part of treatment, making employment of the hybrid theatre essential for patients undergoing LITT-treatment. Additionally, perioperative movement could result in devastating injuries, should the laser catheter be displaced during surgery. Furthermore, the iMRI offers a unique opportunity to detect intraoperative events such as acute hematomas, as demonstrated in our study.

Total median time of surgery was 445 min in the entire study population, comparable to results by Mohammadi et al. and Senft et al. [11, 48]. In our experience, this resulted in only one surgery a day being performed in the hybrid theatre, in comparison to an average of two surgeries a day in the conventional neurosurgical operating suites. Furthermore, as the hybrid operating theatre requires one additional staff member, the safety nurse, compared to conventional surgeries, a higher demand on staff scheduling is anticipated. The fact that the iMRI requires increased personnel resources cannot be overlooked and should be considered when introducing this type of technique in clinical practice. In addition, the IMRIS suite at our facility, due to logistical purposes during implementation, is located at an independent site distant from the conventional operating theatres, compromising the flexibility of circulating nurses and staff. Therefore, the hybrid suite consumes more staff, both in an explicit and in a practical manner, which was a perceived as the main limitation for accessibility at our facility. An iMRI suite located in closer proximity to the neurosurgical operation ward would possibly enable further utilization and a more flexible work flow, resulting in improved care for the individual patient.

Out of the 30% of patients who suffered from postoperative complications in our study, the majority sustained grade Ia (no drugs) or Ib (drugs) complications. No surgeries resulted in death during the early postoperative period of 30 days. We observed permanent postoperative neurological deficits in 13% of patients, in accordance with previous publications by Hatiboglu et al. describing a permanent neurological deficit rate of 9% with iMRI guidance for GBM surgery [18]. A review article by Fugate et al. proclaims that the general complication rate of neurosurgical procedures is roughly 14% [15], indicating that our numbers are aligned with common complication rates for these high-risk surgeries. The frequency of postoperative permanent neurological deficits was irrespective of whether resection was continued or not, (*p* = 0.67), in agreement with findings by Kuhnt et al. [23]. As permanent postoperative neurological deficits were scarce across the entire study population, any final conclusions should be interpreted with a degree of caution. Preliminary implications, however, support the notion that the benefit of extended resection after iMRI in case of remaining pathological tissue outweighs this risk, as the risk of severe complications were comparable. The most prevalent permanent postoperative neurological impairments demonstrated in our study were visual deficits and cranial nerve deficits, where 7 out of 10 (70%) and 3 out of 4 (75%) of deficits were permanent, respectively. Thus, surgeries involving these structures warrant special cautiousness, and may be considered of particular importance to safely perform with the aid of iMRI, as correlating complications to a great extent tend to be permanent.

### Strengths and limitations

Considering that this is a single center study focusing on the surgical aspects and utilization of the iMRI, the sample size is somewhat small. Furthermore, the heterogeneity of the lesions does not allow multivariable subgroup analyses. Since patients were assessed by anesthesiologists and neurosurgeons to evaluate fitness for surgery, a pre-operational selection was inevitable. Additionally, 16 patients, mainly pediatric patients with caregivers, declined participation or could not be included due to other circumstances. Age should therefore be slightly skewed towards older ages in the whole cohort. However, it encompasses 100 out of the 116 first patients undergoing surgery, adequately depicting a startup period of such a facility. An inherent limitation of this study is the retrospective design and its unmatched investigation group. Nevertheless, the large catchment area of our institution ensures a representative sample of neurosurgical patients during a four-year period, as there is no referral bias in the region.

- The iMRI was utilized for tumor surgeries, particularly adult low-grade gliomas and pediatric tumors, as well as for epilepsy surgery and DBS.
- In this heterogenous population, iMRI facilitated continuation of surgery in 50%, primarily due to the presence of residual tumor tissue (90.5%).
- To establish the benefit in maximizing the extent of resection in these brain pathologies future studies are recommended

## Conclusion

Herein, we demonstrate clinical, surgical and radiological variables from the first 100 patients undergoing iMRI utilized neurosurgeries at our facility since introduction in 2017. Indications for surgery differed between pediatric and adult patients. Utilization of iMRI appears to be most frequently applied in tumor surgeries, particularly in pediatric patients, as well as in epilepsy surgery and DBS. Future studies of patients with these particular pathologies are recommended to establish conclusive clinical significance and identify the “essential need” for iMRI.

## Data Availability

Data and materials related to this study are available from the corresponding author upon reasonable request. Restrictions apply to the availability of these data, which were used under license for the current study, and so are not publicly available. However, access may be granted by the authors on a case-by-case basis.
